# Study on the correlation and clinical significance of T-lymphocyte Subsets, IL-6 and PCT in the severity of patients with sepsis

**DOI:** 10.12669/pjms.39.1.5711

**Published:** 2023

**Authors:** Qian Li, Wenwen Yan, Sha Liu, Hui Li

**Affiliations:** 1 Qian Li, Department of Critical Care Medicine, Baoding No.1 Central Hospital, Baoding 071000, Hebei, P.R. China; 2 Wenwen Yan, Department of Critical Care Medicine, Baoding No.1 Central Hospital, Baoding 071000, Hebei, P.R. China; 3 Sha Liu, Department of Critical Care Medicine, Baoding No.1 Central Hospital, Baoding 071000, Hebei, P.R. China; 4 Hui Li, Department of Critical Care Medicine, Baoding No.1 Central Hospital, Baoding 071000, Hebei, P.R. China

**Keywords:** T lymphocyte subsets, IL-6, PCT, Sepsis, Diagnosis

## Abstract

**Objective:**

To evaluate the correlation and clinical significance of T lymphocyte subsets, IL-6 and PCT in the severity of patients with sepsis.

**Methods:**

One-hundred and twenty patients with sepsis admitted to Baoding No.1 Central Hospital from March 05, 2021 to March 05, 2022 were selected and divided into three groups according to the severity of the disease: the sepsis group, the severe sepsis group and the septic shock group, with 40 cases in each group. The venous blood of all patients was drawn with a sterile vacuum blood collection tube after admission to detect the levels of T lymphocyte subsets CD3+, CD4+, CD8+, CD4+/CD8+, and the venous blood was collected to detect the levels of interleukin-6 (IL-6) and procalcitonin (PCT). The three groups of patients were compared to analyze whether there were differences, and whether there was a correlation between the level of each indicator and the prognosis of patients after treatment.

**Results:**

The levels of CD3+, CD4+ and CD4+/CD8+ in the three groups decreased with the aggravation of the disease, with a significant difference (p=0.00). The levels of IL-6 and PCT increased with the aggravation of the disease among the three groups, with statistically significant differences (IL-6, p=0.00; PCT, p=0.01). The better the patients recovered after treatment, the higher the levels of CD4+ and CD4+/CD8+, and the two were positively correlated; While the lower the levels of IL-6 and PCT, the two were negatively correlated.

**Conclusion:**

Peripheral blood T lymphocyte subsets and serum IL-6, PCT are abnormally expressed in patients with sepsis, and have a close bearing on the severity of the disease, which has a certain predictive value for patients after recovery. In view of this, the above indicators are of high clinical significance.

## INTRODUCTION

Sepsis, as a common acute critical disease in the intensive care unit (ICU), is characterized by rapid disease progression, serious consequences and high mortality.^1^ It can be divided into sepsis, severe sepsis and septic shock according to the severity of the disease.^2^ The pathogenesis of sepsis is associated with a variety of factors, such as immune dysfunction, endotoxin translocation and inflammatory factor secretion.^3^ Clinically, the methods commonly used in the treatment of sepsis include antibiotic therapy, fluid resuscitation, hormone therapy, and maintenance of blood supply to important organs.^4^ However, specific guidelines are still lacking in the judgment of the therapeutic effect and prognosis of the disease, and how to judge the severity and prognosis of the disease is extremely important in clinical work. Studies have concluded that^5^ that the level of lymphocyte subsets in peripheral blood of patients with sepsis is closely related to their prognosis. PCT has high clinical application value in sepsis, and can be used as a reliable indicator for the diagnosis of sepsis.^6^ According to Patel and his colleagues^7^, the levels of IL-6, IL-8, IL-10 and other inflammatory factors in patients with sepsis were all increased compared with those in healthy individuals, and the levels of these markers were correlated with the severity of sepsis to a certain extent. Based on this, patients with sepsis were enrolled in this study to analyze the correlation between the changes of T lymphocyte subsets, PCT, IL-6 and the severity of the disease, so as to provide a reliable reference for clinical treatment and improvement of prognosis.

## METHODS

A total of 120 patients with sepsis admitted to Baoding No.1 Central Hospital from March 05, 2021 to March 05, 2022 were selected as the study group and divided into three groups according to the severity of the disease: the sepsis group, the severe sepsis group and the septic shock group, with 40 cases in each group. Among them, 17 males and 23 females were grouped into the sepsis group, aged 30-67 years old, with an average of 55.74 ± 9.76 years old. Nineteen males and 21 females were grouped into the severe sepsis group, aged 30-65 years old, with an average of 54.13 ± 11.32 years old. 13 males and 27 females were grouped into the septic shock group, aged 35-70 years old, with an average of 55.89 ± 10.19 years old. There was no significant difference in general data between the three groups, which were comparable (Table-I). Meanwhile, 80 healthy subjects were selected as the control group, and the baseline data of the two groups were balanced, which were comparable (p>0.05).

**Table I:** Comparative analysis of general data of the three groups (*χ̅*±*S*) n=40.

Indicators	Sepsis group	Severe sepsis group	Septic shock group	F/χ^2^	P
Age (years old)	54.74±9.76	54.13±11.32	55.89±10.19	0.52	0.61
Male (%)	17 (42.5%)	19(47.5%)	13 (32.5%)	1.87	0.17
***Infection sites***					
Abdominal cavity	13 (32.5%)	15 (37.5%)	11 (27.5%)	0.22	0.63
Soft tissue	10 (25%)	7 (17.5%)	7 (17.5%)	0.67	0.41
Urinary tract	12 (30%)	14 (35%)	15 (37.5%)	0.50	0.46
Lungs	5 (12.5%)	1 (2.5%)	4 (10%)	2.88	0.09
Other sites	0 (0%)	3 (7.5%)	3 (7.5%)	3.11	0.07

P>0.05.

### Ethical approval:

The study was approved by the Institutional Ethics Committee of Baoding No.1 Central Hospital(No.: [2021]068; Date: May 27, 2021), and written informed consent was obtained from all participants.

### Inclusion criteria:

- All patients who met the diagnostic criteria for sepsis and were confirmed by clinical symptoms, laboratory examination and microbiological examination, etc.^8^;
- Patients under the age of 70;
- Patients with no previous history of infectious diseases;
- Patients whose family members are willing and able to cooperate to complete the study;
- Patients who signed the informed consent.

### Exclusion Criteria:

- Patients complicated with severe organic or congenital diseases of heart, liver and kidney;
- Patients whose hospital stay is less than 24 hour and unable to complete the study
- Patients with incomplete clinical data;
- Patients with mental system diseases who cannot cooperate with the completion of the study;
- Patients who have recently taken relevant drugs such as immunosuppressants that affect the study in the near future.

Five ml of venous blood was drawn with a sterile vacuum blood collection tubes for all patients after admission, flow cytometry (BD Company, USA) was used to detect the levels of T lymphocyte subsets CD3+, CD4+, CD8+, CD4+/CD8+, and venous blood was collected to detect the levels of interleukin-6 (IL-6) and procalcitonin (PCT) (ELISA method, kit provided by French R&D Systems). The three groups of patients were compared to analyze whether the levels of CD3+, CD4+, CD8+, CD4+/CD8+, PCT and IL-6 were correlated with the prognosis of patients after treatment. All blood samples were labeled according to the sepsis group, the severe sepsis group and the septic shock group, and were performed by two senior laboratory physicians in strict accordance with the operating procedures. The results were judged by two senior physicians.

*Observation indicators:* 1) The similarities and differences of the levels of T cell subsets, IL-6 and PCT between the study group and the control group were compared and analyzed; 2) The levels of T lymphocyte subsets in the sepsis group, the severe sepsis group and the septic shock group were compared and analyzed; 3) The levels of IL-6 and PCT in the sepsis group, the severe sepsis group and the septic shock group were compared and analyzed; 4) The correlation between different groups in terms of the levels of T cell subsets, IL-6, and PCT after recovery were compared and analyzed. Judgment criteria after recovery: One week after treatment, patients were evaluated according to their symptoms and laboratory examination^9^. Criteria were divided into improved, stable and deteriorated.

### Statistical Analysis:

All the data were statistically analyzed by SPSS 20.0 software, and the measurement data was expressed as (*χ̄*±*S*). Repeated measurement analysis of variance was used for comparison of test data, LSD-t test was used for pairwise comparison, and 2-test was adopted for rate comparison. Two independent samples t test was used for comparison between groups, and the correlation was expressed by the Pearson correlation coefficient. P<0.05 indicates a statistically significant difference.

## RESULTS

The levels of CD3+, CD4+ and CD4+/CD8+ in the study group were significantly lower than those in the health control group, with a significant difference (p<0.05), while the levels of PCT and IL-6 were significantly higher than those in the health control group, with a statistically significant difference (p<0.05) (Table-II).

**Table-II:** Comparative analysis of the levels of T cell subsets, IL-6 and PCT between the study group and the control group (*χ̅*±*S*).

Group	n	CD3^+^(%)*	CD4^+^(%)*	CD8^+^(%)	CD4^+^/CD8^+^*	PCT(ng/ml)*	IL-6(ng/L)*
Study group	120	40.27±6.83	27.79±7.26	21.77±5.03	1.31±0.49	0.57±0.08	14.07±2.15
Control group	80	62.13±7.21	37.62±5.87	23.35±6.82	1.49±0.58	0.15±0.04	8.97±2.24
t/χ^2^		21.68	10.10	1.88	2.36	43.45	16.16
p		0.00	0.00	0.06	0.02	0.00	0.00

* p<0.05.

The changes of T lymphocyte subsets among the three groups are shown in Table-III, indicating that the levels of CD3+, CD4+ and CD4+/CD8+ in the three groups decreased with the aggravation of the disease, with significant differences(p=0.00). However, there was no significant change in the level of CD8+ among the three groups(p=0.25).

**Table-III:** Comparative analysis of changes in the level of T lymphocyte subsets among the three groups (*χ̅*±*S*) n=40.

Indicators	Sepsis group	Severe sepsis group	Septic shock group	F	P
CD3^+^(%)	47.36±9.34	40.74±8.22	32.42±7.45	7.96	0.00
CD4^+^(%)	35.15±6.27	28.61±5.34	23.28±6.31	8.44	0.00
CD8^+^(%)	20.27±4.81	20.74±3.75	21.43±4.21	1.15	0.25
CD4^+^/CD8^+^	1.48±0.43	1.35±0.67	1.21±0.52	4.59	0.00

*p<0.05

The levels of IL-6 and PCT among the three groups increased with the aggravation of the disease, and the difference between the groups was statistically significant (IL-6, p=0.00; PCT, p=0.01) (Table-IV).

**Table-IV:** Comparative analysis of the levels of IL-6 and PCT between the three groups (*χ̅*±*S*) n=40.

Indicators	Sepsis group	Severe sepsis group	Septic shock group	F	P
IL-6(ng/L)	9.78±2.57	14.76±1.27	17.54±4.78	9.04	0.00
PCT(ng/ml)	0.37±0.13	0.52±0.24	0.68±0.04	2.61	0.01

P<0.05.

The level of T lymphocyte subsets was correlated with the recovery of sepsis. The better the patients recovered after treatment, the higher the levels of CD4+ and CD4+/CD8+, and the two were positively correlated, while CD3+ and CD8+ have no obvious correlation with the recovery of sepsis (Table-V). The levels of PCT and IL-6 were correlated with the recovery of sepsis. The lower the level of PCT and IL-6, the better the recovery, showing a negative correlation (Table-VI).

**Table-V:** Correlation between the levels of T cell subsets and the recovery of different sepsis (*χ̅*±*S*) n=40.

Criteria after recovery	CD3^+^(%)	CD4^+^(%)	CD8^+^(%)	CD4^+^/CD8^+^
Deteriorated	0.13	0.35	0.16	0.27
Stable	0.12	0.52	0.14	0.31
Improved	0.15	0.61	0.18	0.43

**Table-VI:** Correlation between the levels of PCT and IL-6 cell subsets and the recovery of different sepsis (*χ̅*±*S*) n=40.

Criteria after recovery	PCT	IL-6
Deteriorated	-0.62	-0.43
Stable	-0.43	-0.31
Improved	-0.15	-0.26

## DISCUSSION

Sepsis, as a common serious infectious disease, has become a common cause of death in critically ill patients. its pathogenesis is the imbalance of the dynamic balance between inflammation and pro-inflammatory reaction, which leads to immune dysfunction of the body, and then induces the body to produce inflammatory factors and inflammatory mediators, which may eventually lead to insufficient tissue perfusion, organ dysfunction, etc.^10^ Patients with sepsis are initially characterized as lack of specificity clinically, most of whom have atypical symptoms at the early stage of the disease, but can progress within a few hours, seriously threatening the lives of patients. It has been confirmed by studies that the unclear diagnosis of patients with severe sepsis within the initial six hours is the main risk factor for the increased mortality of patients with sepsis.^11^ Early diagnosis and treatment of sepsis is the key factor to reduce mortality and improve the prognosis of patients. However, opportunities for optimal treatment for patients are often missed due to the lack of specific indicators for early diagnosis of sepsis and the long duration of blood culture results. Consequently, a favourable detection index should be high sensitivity, earlier appearance, and have a certain predictive value for patients after recovery, which can be used for early diagnosis and treatment guidance of patients.

According to animal experimental studies^12^, abnormal immune regulation of the body plays an important role in the occurrence and development of sepsis. T lymphocytes are the main defense cells of the body against infection. It was believed by Lei et al.^13^ That sepsis and infection could lead to a decrease in the percentage of blood T lymphocytes. It was indicated in the study by Wu et al.^14^ That the core role was played by the apoptosis or reduction in the number of CD4+ T cells in the progression of sepsis, and the induction and activation of CD4+ and the improvement of its level may play a certain role in the prognosis of sepsis. In addition, the study of Francois and his colleagues^15^ also suggested that the reduction of CD4+ and CD8+ immune effects might be the key mechanism for the development and mortality of sepsis. According to the study of Huang et al.^16^ CD4+ T cells in patients with sepsis were significantly higher than those in the health control group. The percentage of CD4+ T cells can predict the outcome of patients with sepsis It was confirmed in our study that the levels of CD3+, CD4+ and CD4+/CD8+ in patients with sepsis were significantly lower than those in the health control group, with a significant difference (p<0.05). The levels of CD3+, CD4+ and CD4+/CD8+ have decreased with the exacerbation of the disease, and the difference was significant (p=0.00). The higher the levels of CD4+ and CD4+/CD8+, the better the recovery of patients after treatment, showing a positive correlation, suggesting that there is an abnormal immune regulation in patients with sepsis. T lymphocyte subsets can better reflect the severity of the disease, especially the levels of CD4+ and CD4+/CD8+, which are not only correlated with the severity of the disease, but also have a certain bearing on the outcome of the disease.

PCT is secreted by thyroid C cells. In healthy people, the level of serum PCT is considerably low. In contrast, in local infection, viral infection and autoimmune diseases, the level of PCT is slightly increased or not increased, and PCT is released in large quantities under the promotion of inflammatory factors in the body when the body is seriously infected with bacteria or sepsis. Moreover, the level of PCT increased further with the aggravation of the disease, and decreased with the improvement of the disease. It can therefore be seen that PCT has good distinguishing characteristics to distinguish bacterial inflammation from viral inflammation and obtain results quickly.^17^ Besides, the level of PCT has a close bearing on the degree of inflammatory reaction in sepsis, and the dynamic detection of PCT level is conducive to evaluating the changes in the condition of patients with sepsis. Studies have suggested that the level of serum PCT is closely linked to the severity grading and disease development trend of sepsis.^18^ Interleukin 6 (IL-6), as a commonly used serum marker for the evaluation of inflammatory diseases^19^, can be used to distinguish severe patients suspected of infection.^20^

It was concluded by Ma et al.^21^ That IL-6 and PCT had similar values in the diagnosis of sepsis, but the value of the former was slightly higher than that of CRP. However, according to Song et al.^22^, IL-6 was superior to PCT in terms of diagnostic and prognostic value for sepsis and septic shock. It was considered by Memar et al.^23^ That IL-6 is the most effective marker for evaluating the prognosis of sepsis, and can monitor and guide the application of antibiotics. As indicated in a completely randomized controlled study^24^, patients with abnormal IL-6 had a higher 60 day mortality rate. According to Thao et al.^25^, a reduction of the level of IL-6 ≥ 86% within 24h after admission was a survival predictor for patients with sepsis and septic shock in the population.

It was suggested in our study that the levels of PCT and IL-6 were significantly higher than those in the health control group (p<0.05). The levels of IL-6 and PCT increased with the aggravation of the disease (IL-6, p=0.00; PCT, p=0.01). The levels of PCT and IL-6 were correlated with the recovery of sepsis, that is, the lower the levels of PCT and IL-6, the better the patients recovered after treatment, showing a negative correlation.

### Limitations of the study:

It includes fewer patients were included in the study, and only the indicators of patients with different severity after admission are compared and analyzed, without more systematic post-treatment review and follow-up. In addition, the guiding significance of each indicator for the treatment and the judgment of the effect are not involved in this study. In view of this, proactive countermeasures are being taken to further improve the data, increase the number of samples and dynamically review the changes of various indicators after treatment, so that the study can be further enriched, and the follow-up can be increased, hereby demonstrating the guiding value of various indicators involved in the study for treatment

## CONCLUSION

Peripheral blood T lymphocyte subsets and serum IL-6, PCT are abnormally expressed in patients with sepsis, and have a close bearing on the severity of the disease. Some indicators can have certain predictive value for the recovery of patients. In view of this, the above indicators are of high clinical significance.

### Authors’ Contributions:

**QL &** **WY:** Designed this study and prepared this manuscript,and are responsible and accountable for the accuracy or integrity of the work.

**SL:** Collected and analyzed clinical data.

**HL:** Data analysis**,** significantly revised this manuscript.
